# Age-Dependent Metabolic Profiles Unravel the Metabolic Relationships within and between Flax Leaves (*Linum usitatissimum*)

**DOI:** 10.3390/metabo10060218

**Published:** 2020-05-26

**Authors:** Nicole Pontarin, Roland Molinié, David Mathiron, Job Tchoumtchoua, Solène Bassard, David Gagneul, Benjamin Thiombiano, Hervé Demailly, Jean-Xavier Fontaine, Xavier Guillot, Vivien Sarazin, Anthony Quéro, François Mesnard

**Affiliations:** 1 UMR 1158 Transfontalière BioEcoAgro, BIOlogie des Plantes et Innovation (BIOPI), UPJV, Faculté de Pharmacie, 1 rue des Louvels, 80025 Amiens CEDEX, France; Nicole.Pontarin@anu.edu.au (N.P.); roland.molinie@u-picardie.fr (R.M.); Job.Tchoumtchoua@celabor.be (J.T.); solene.bassard@u-picardie.fr (S.B.); jean-xavier.fontaine@u-picardie.fr (J.-X.F.); 2 PFA, UPJV, 33 rue Saint Leu, 80039 Amiens, France; david.mathiron@u-picardie.fr; 3 Biomass Valorization Platform—Extraction Department, CELABOR, Avenue du Parc 38, 4650 Herve, Belgium; 4 UMR 1158 Transfontalière BioEcoAgro, Institut Charles Viollette (ICV), Université de Lille, Cité Scientifique, 59655 Villeneuve d’Ascq, France; david.gagneul@univ-lille1.fr; 5 Swammerdam Institute for Life Sciences, University of Amsterdam, Science Park 904, 1098 XH Amsterdam, The Netherlands; b.thiombiano@uva.nl; 6 CRRBM, UPJV, 33 rue Saint Leu, 80039 Amiens, France; herve.demailly@u-picardie.fr; 7 Laboulet Semences, 6 rue du Capitaine N’Tchorere, 80270 Airaines, France; xguillot@laposte.net; 8 SADEF-AgroStation, 30 rue de la Station, 68700 Aspach-Le-Bas, France; sarazinv@agrostation.fr

**Keywords:** flax, primary metabolites, specialized metabolites, leaf developmental stages, LC-MS, GC-MS

## Abstract

Flax for oil seed is a crop of increasing popularity, but its cultivation needs technical improvement. Important agronomic traits such as productivity and resistance to stresses are to be regarded as the result of the combined responses of individual organs and their inter-communication. Ultimately, these responses directly reflect the metabolic profile at the cellular level. Above ground, the complexity of the plant phenotype is governed by leaves at different developmental stages, and their ability to synthesise and exchange metabolites. In this study, the metabolic profile of differently-developed leaves was used firstly to discriminate flax leaf developmental stages, and secondly to analyse the allocation of the metabolites within and between leaves. For this purpose, the concentration of 52 metabolites, both primary and specialized, was followed by gas chromatography (GC-) and liquid chromatography coupled to mass spectrometry (LC-MS) in alternate pairs of flax leaves. On the basis of their metabolic content, three populations of leaves in different growth stages could be distinguished. Primary and specialized metabolites showed characteristic distribution patterns, and compounds similarly evolving with leaf age could be grouped by the aid of the Kohonen self-organising map (SOM) algorithm. Ultimately, visualisation of the correlations between metabolites via hierarchical cluster analysis (HCA) allowed the assessment of the metabolic fluxes characterising different leaf developmental stages, and the investigation of the relationships between primary and specialized metabolites.

## 1. Introduction

Metabolism, intended as the extensive net of metabolites and their enzymatic reactions, defines the phenotype of any living unit, from cells to whole organisms. Because metabolites are the end products of gene expression, metabolic profiling provides the most accurate description of the physiological state of an organism. However, when studying the metabolic phenotype, pooling the different levels of organisation of an organism (organs, tissues, cell types) may result in undesirable dilution of site specific up- or down-accumulated metabolites [1]. Spatial resolution of the metabolome is thus of fundamental importance for a correct appraisal of biological processes [2]. In plants, an additional degree of complexity is the result of the fact that identical organs coexist on the same plant with different degrees of development. On the metabolic point of view, each developmental stage corresponds to a specific physiological stage characterised by distinctive metabolic processes. For example, in the newly-formed leaves, the main physiological process is growth; on the contrary, while development proceeds, the growth rate decreases and other physiological functions take over, such as photosynthesis. However, metabolic profiles characteristic of a given stage of development are also shaped by the inter-relationships between differently developed organs. Growth of the new leaves, for example, is supported by fully-developed leaves [3], which supply the carbohydrates produced through photosynthesis. In turn, the demand for photosynthates is established by the new leaves themselves through regulation of cell osmotic potential and plant water fluxes [4,5]. In this sense, spatially resolved metabolic studies can provide information on processes going beyond the physiological state of the single organ. This dynamic aspect of the metabolome is of major importance when studying plant plastic [6] and adaptive responses; from predation to drought, all of the responses of plants to a given stimulus are driven by the allocation of metabolites within and between organs of different types or at different stages of development [7]. Generally, the regulation of primary metabolite allocation affects fundamental processes like growth and development. Other processes aimed at increasing plant fitness in a specific environment are rather related to specialized metabolism, such as protection against herbivores and pathogens, reduction of oxidative stress, or competition against other plants [8]. In addition, as primary metabolites constitute the bricks for the synthesis of specialized metabolites, the latter intimately depends on the availability, the import, or the remobilisation of the former ones [9]. This, in fact, determines the biosynthetic cost of a specialized metabolite [10]. For these reasons, the content of primary and specialized metabolites and their relationships represent a key in the interpretation of physiological, developmental, and adaptive processes.

Oilseed flax is a developing culture thanks to its desirable nutraceutical properties [11]. For the first time, we have recently characterized the major phenolic compounds of flax leaves [12]. In the present work, we describe how primary and specialized metabolites are distributed in the shoot of flax plants. Their concentrations were followed in leaves according to the leaf position along the growth axis. Because the evolution of the primary and specialized metabolites was monitored so closely, the experimental design was ideal to discuss the relationships between primary and specialized metabolites in populations of leaves at different developmental stages. This work will constitute the basis for further understanding of how the relationships between primary and specialized metabolism are modified in different physiological conditions relative to the degree of leaf development [13]. Ultimately, the aim will be to bring to light novel key metabolic hubs at the interface between primary and specialized metabolism in order to improve crop quality traits [14].

## 2. Results

### 2.1. Growth Parameters of Three Flax Leaf Populations at Different Stages of Development

**Figure 1:**
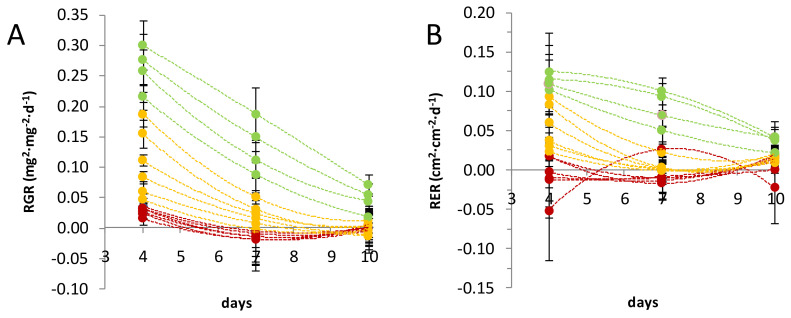
Relative growth rate (RGR) and relative expansion rate (RER) of leaf populations. Leaf RGR (**A**) and RER (**B**) trends during a 10-day timeframe were used to discriminate leaf stages of development. Leaf samples (dots) are ordered following their numerical sequence. From the top to the bottom, leaves: 59–60, 57–58, 55–56, 51–52, 47–48, 43–44, 39–40, 35–36, 31–32, 27–28, 23–24, 19–20, 15–16, 11–12, 7–8, and 3–4. Three different populations of leaves were defined as young (green), transition (yellow), and mature (red). RGR and RER values were calculated between days 0 and 4, 4 and 7, and 7 and 10. For every leaf sample, n = 6.

Before studying the distribution of the metabolites in flax leaves, data on the physiology of the leaves were obtained by measuring the leaf structural and expansive growth rate (RGR: relative growth rate, RER: relative expansion rate) in a 10-day time span. Leaves were numbered from the bottom (oldest leaves) to the top (youngest leaves). Two consecutive leaves were pooled. Each sample corresponds to the combination of leaves of two individual plants. Some leaf pairs were omitted (see Supplementary Materials 1). As expected, because leaf growth follows an exponential trend, three populations of leaves were discriminated: leaves 51–52 to 59–60 were in the exponential phase of their structural and expansive growth, leaves 27–28 to 47–48 situated in the descendant phase of the growth curve, and leaves 3–4 to 23–24 were characterised by no structural and expansive growth (Figure 1). The populations were identified as apical young leaves (YLs), intermediate transition leaves (TLs), and basal mature leaves (MLs), respectively. In the timeframe 0–4 days, TL and YL displayed RGR values of 0.04–0.19 and 0.21–0.30, respectively, while RER values were of 0.02–0.10 and 0.10–0.13, respectively. From day 4 to 7, TL had RGR values lower than 0.06, while YL had RGR values between 0.08 and 0.19. RER values were equivalent or close to 0 for TL and comprised between 0.05 and 0.11 for YL. Between 7 and 10 days, YL showed RGR values of 0.02–0.08 and RER values of 0.02–0.05, while TL RGR and RER values were both equivalent or close to 0. In all these time frames, ML presented RGR and RER values equivalent or close to zero.

### 2.2. Age-Dependent Distribution of Flax Leaf Metabolites

**Figure 2:**
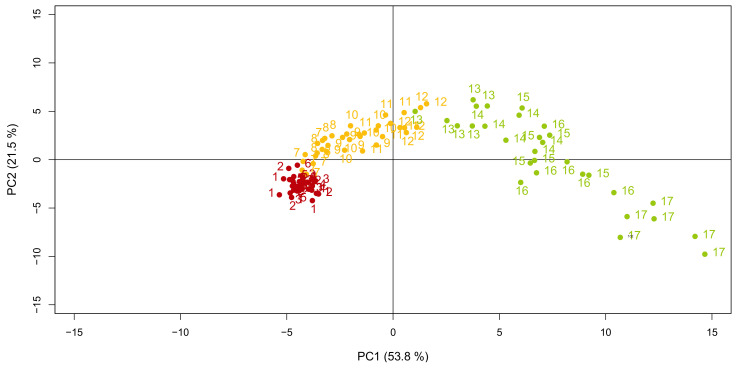
Principal component analysis (PCA) showing the distribution of flax leaves based on their metabolic content. The samples are identified as follows: 1: leaves 3–4; 2: leaves 7–8; 3: leaves 11–12; 4: leaves 15–16; 5: leaves 19–20; 6: leaves 23–24; 7: leaves 27–28; 8: leaves 31–32; 9: leaves 35–36; 10: leaves 39–40; 11: leaves 43–44; 12: leaves 47–48; 13: leaves 51–52; 14: leaves 55–56; 15: leaves 57–58; 16: leaves 59–60; 17: apical bunch of leaves, from leaf 61. The samples are differently coloured whether they belong to the mature (red), the transition (yellow), or the young (green) populations of leaves, as defined in Figure 1. For every leaf sample, n = 6.

**Figure 3:**
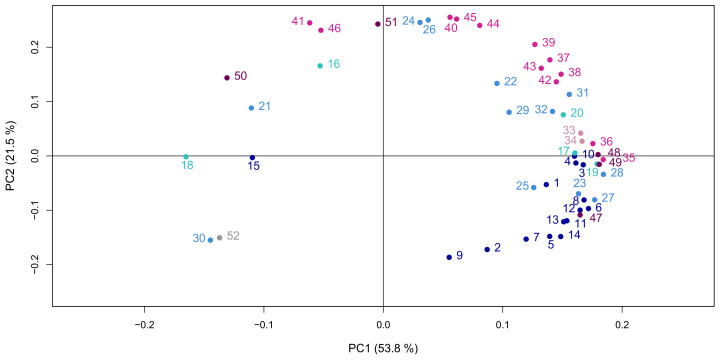
PCA showing the loading plot of the metabolites. The metabolites are coloured with respect to their chemical identity: amino acids and derivatives (dark blue), carbohydrates and derivatives (light blue), tricarboxylic acids (teal), cyanogenic glycosides (pink), flavonoids (fuchsia), coniferyl alcohol derivatives (plum), and inorganic compounds (grey). The metabolites are identified as follows: 1: alanine; 2: aspartate; 3: glutamate; 4: glutamine; 5: glycine; 6: isoleucine; 7: leucine; 8: methionine; 9: phenylalanine; 10: proline; 11: tryptophan; 12: valine; 13: β-alanine; 14: ethanolamine; 15: γ-aminobutyric acid (GABA); 16: citrate; 17: fumarate; 18: malate; 19: succinate; 20: α-ketoglutarate (α-KG); 21: [erythreonate]; 22: fructose; 23: glucose; 24: glycerate; 25: G6P; 26: myo-inositol; 27: quinate; 28: shikimate; 29: sucrose; 30: tartrate; 31: [threonate]; 32: xylose; 33: linamarin; 34: lotaustralin; 35: vitexin; 36: isovitexin; 37: vicenin-2; 38: vicenin-1; 39: schaftoside; 40: swertisin; 41: triticuside A; 42: isoorientin; 43: orientin; 44: lucenin-2; 45: carlinoside; 46: swertiajaponin; 47: lariciresinol-4-*O*-glucoside (LMG); 48: pinoresinol-4-*O*-glucoside (PMG); 49: pinoresinol diglucoside (PDG); 50: 3-Hydroxy-2-[4-[(1*E*)-3-hydroxy-1-propen-1-yl]-2-methoxyphenoxy]propyl-β-d-glucoside (HHMPG); 51: dehydro-diconiferyl alcohol-4-*O*-glucoside (DCG); 52: inorganic phosphate (Pi).

In order to see if the three populations of flax leaves defined above could also be distinguished on the basis of their metabolic content, a set of fifty-two metabolites were analysed by gas chromatography (GC-MS) and liquid chromatography coupled to mass spectrometry (LC-MS). This included fifteen amino acids and derivatives, five tricarboxylic acids, twelve carbohydrates and derivatives, two cyanogenic glycosides, three coniferyl alcohol derivatives, fourteen flavonoids, and one inorganic compound (inorganic phosphate, Pi). The principal component analysis (PCA) was afterward used to visualise the distribution of the leaves based on their metabolic composition. The first two principal components (PCs) explained 75.3% of the variability between leaves. Three main trends could be observed, each corresponding to the previously identified populations (Figure 2): ML closely grouped together, TL distributed consecutively along the PCs 1 and 2 with respect to their numerical order, while YL (including the apical bunch of leaves, or leaves 61+) also distributed consecutively but in a more scattered fashion. The loading plot was afterward produced to assess the effect of each variable (each metabolite) on the principal components (PC1 and PC2) and, therefore, on the data structure (Figure 3). Loadings can range from −1 to 1. The variable influence on the PCs is stronger when the loading is close to −1 or 1, while it is weaker when the loading is close to 0. Taking this into account, the loading plot of the metabolites showed that all the metabolites were equally discriminating. The spread of the metabolites along PC1 of the loading plot also revealed which metabolites were responsible for the separation of the samples according to their metabolic composition [15]. Forty-two out of the fifty-two analysed compounds discriminated the leaf samples of the YL population; within them, all the amino acids except γ-aminobutyric acid (GABA) distinguished the apical bunch of leaves, while the remaining YLs were discriminated by the majority of the compounds of the other classes. Just one tricarboxylic acid (citrate), two flavonoids (triticuside A and swertiajaponin), and one lignan (dehydro-diconiferyl alcohol-4-*O*-glucoside: DCG) characterised the TL samples. GABA, malate, erythreonate, tartrate, inorganic phosphate (Pi), and 3-Hydroxy-2-[4-[(1*E*)-3-hydroxy-1-propen-1-yl]-2-methoxyphenoxy]propyl-β-d-glucoside (HHMPG) discriminated the ML samples.

**Figure 4:**
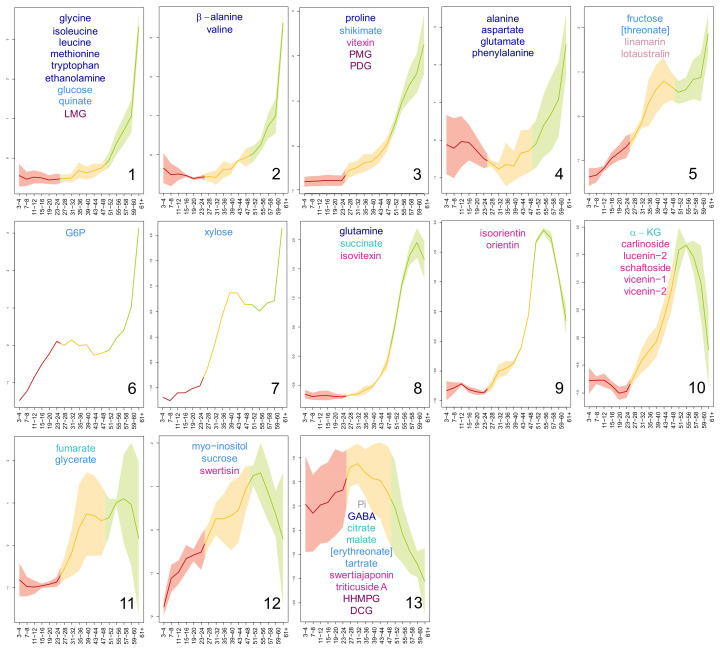
Metabolites grouped on the basis of their age-dependent content evolution in flax leaves. The classes (identified by the number within the circle) were defined using the Kohonen self-organising map (SOM) algorithm. Differently coloured lines represent the content evolution in basal mature leaves (MLs) (red), intermediate transition leaves (TLs) (yellow), and apical young leaves (YLs) (green). The lighter coloured areas represent the standard deviation of the metabolites from the metabolic content of their respective class. The numbers below the x-axis identify the leaf samples. The metabolites are coloured with respect to their chemical identity: amino acids and derivatives (dark blue), carbohydrates and derivatives (light blue), tricarboxylic acids (teal), cyanogenic glycosides (pink), flavonoids (fuchsia), coniferyl alcohol derivatives (plum), and inorganic compounds (grey). For every leaf sample, n = 6.

After the multivariate analysis, univariate analysis was used to follow the content of each metabolite in flax leaves (Supplementary Materials 2). The metabolites showed a characteristic evolution in content throughout the plant, which corresponded to their age-dependent profile. Metabolites whose content showed a similar evolution in flax leaves during their development were grouped using the Kohonen self-organising map (SOM) algorithm (Figure 4). The SOM algorithm is an unsupervised method used to project a high-dimensional input data space into a bi-dimensional lattice matrix in an ordered fashion. The lattice matrix is composed of nodes connected in a network, where each node represents a reference vector; during the SOM establishment, each sample vector is associated to a node using a distance algorithm, so that, at the end of the process, similar sample vectors would be represented by the same node. In this study, thirteen classes of metabolic profiles were identified by applying the SOM algorithm to a 4*5 nodes matrix (Figure 4). The age-dependent content evolution of the classes is dimensionless. Therefore, here, when the metabolic content of a leaf sample is mentioned, its value is meant to be read as a relative value of the highest content assumed by the profile of its class (for more details, see Material and Methods, Section 4.5.3). On the whole, the majority of the metabolites were more concentrated in the YL. The metabolic profiles of the first three classes (1, 2, and 3) peaked in the apical leaves (leaves 61+). Globally, their evolution with leaf age was very similar, showing consistent metabolic contents in ML and content increases in TL and YL. YL increases were greater than the TL increases. In detail, TL content increases were very small in class 1, small in class 2, and moderate in class 3. The intensity of the content increases in YL changed accordingly (class 1 > 2 > 3). The metabolic profiles of class 4 and 5 also peaked in leaves 61+, however, they showed higher average ML contents. Additionally, class 5 metabolites also had substantially higher relative TL values if compared with the previous four classes. Classes 6 and 7 included one metabolite each, G6P and xylose, respectively. The metabolic profile of xylose resembled to that of class 5, however, it showed a lesser ML increase. On the contrary, G6P shared exactly the notable content increases in ML with class 5, but its TL contents were consistent. The profiles of classes 8, 9, and 10 recalled those of classes 1, 2, and 3, particularly for the progressive increase of TL contents, which were, however, greater in value. As a main difference from classes 1, 2, and 3, the profiles of classes 8, 9, and 10 did not peak in the most apical leaves 61+. In detail, class 8 metabolic profile peaked in the sub-apical leaves 59–60, and profiles of classes 9 and 10 peaked in leaves 55–56. Additionally, the metabolic content of leaves 61+ of class 10 was considerably lower relative to that of class 9. The last three classes (11, 12, and 13) grouped metabolites whose age-dependent profiles were more variable (higher standard deviations relative to the other classes) (refer also to the metabolic profiles of Supplementary Materials 2). Nonetheless, these three classes shared higher ML contents relative to the other classes, and TL contents comparable to (classes 12 and 11) or higher than (class 13) YL contents.

Concerning the distribution of the metabolites in these classes with regards to their chemical identity (Figure 4), (i) thirteen of the fifteen amino acids showed apical predominance (peaked in leaves 61+) and belonged to the first four classes, as well as vitexin and the three lignans lariciresinol-4-*O*-glucoside (LMG), pinoresinol-4-*O*-glucoside (PMG), and pinoresinol diglucoside (PDG); (ii) seven of the twelve carbohydrates and derivatives also showed apical predominance and were scattered among the first seven classes, together with the cyanogenic glycosides linamarin and lotaustralin; and (iii) eight out of twelve flavonoids were distributed in classes 8, 9, and 10 and were prevalent in the YL. The rest of the carbohydrates and the specialized metabolites showed a more varied, but generally not-apical distribution, belonging to classes 11, 12, and 13. The distribution of tricarboxylic acids did not follow any specific trends.

### 2.3. Visualisation of the Metabolic Correlations within Leaf Populations

**Figure 5:**
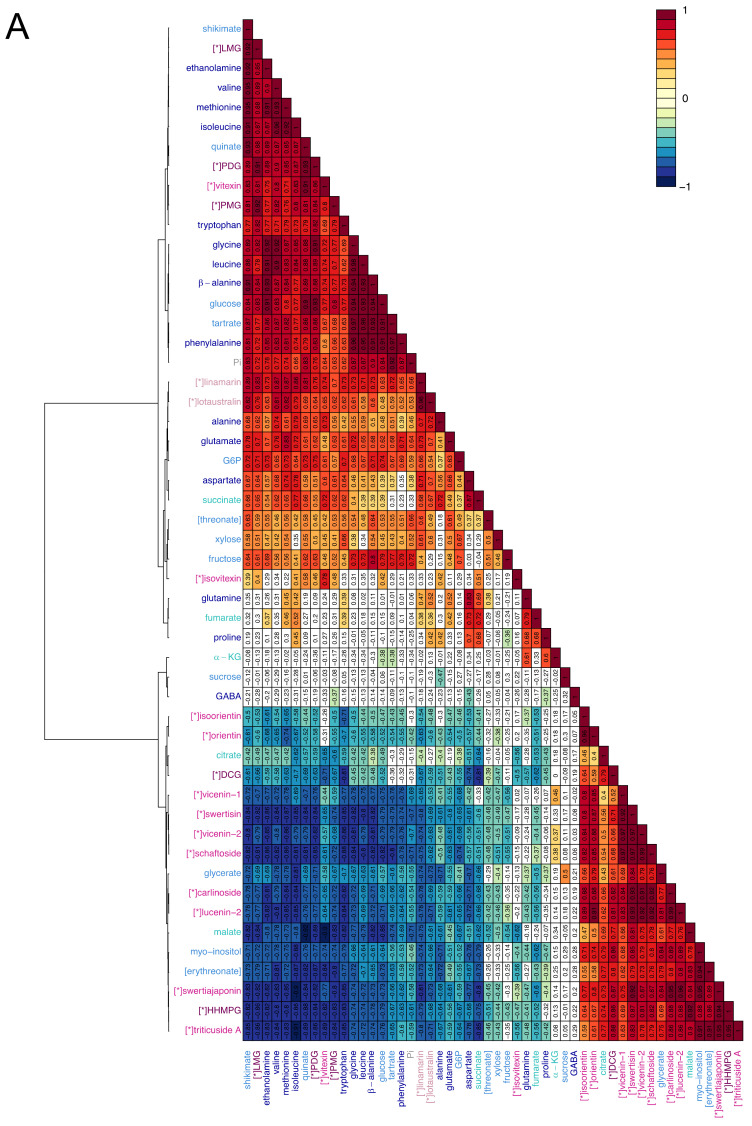

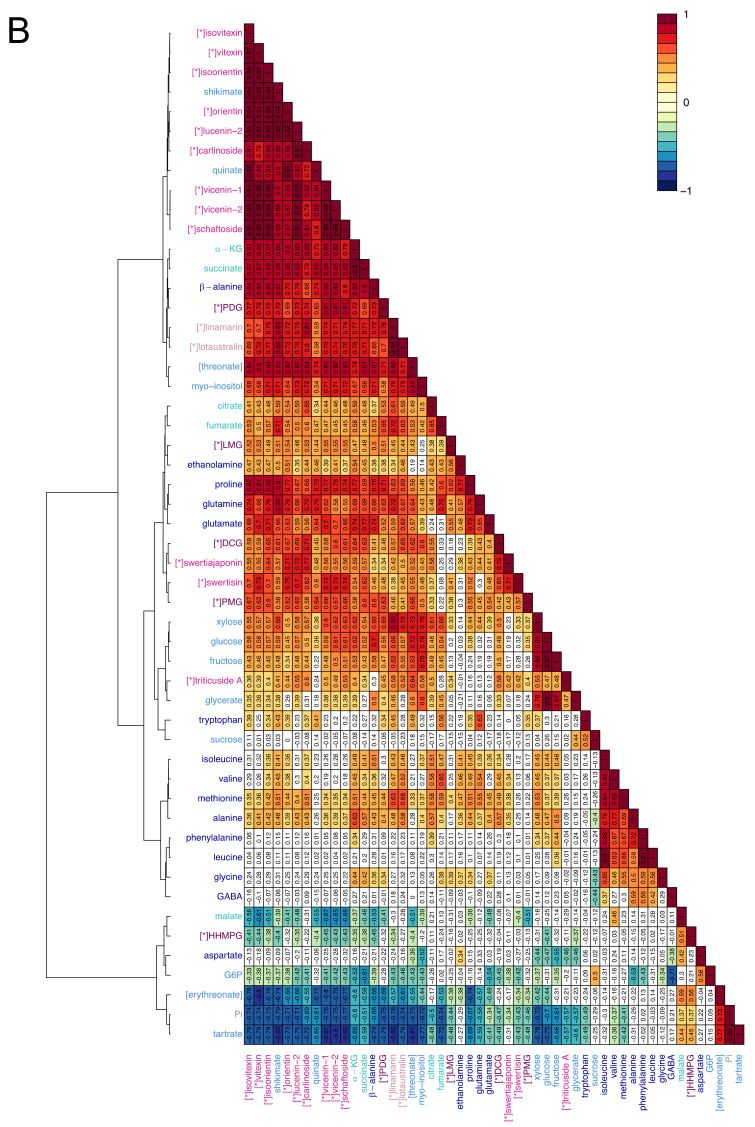

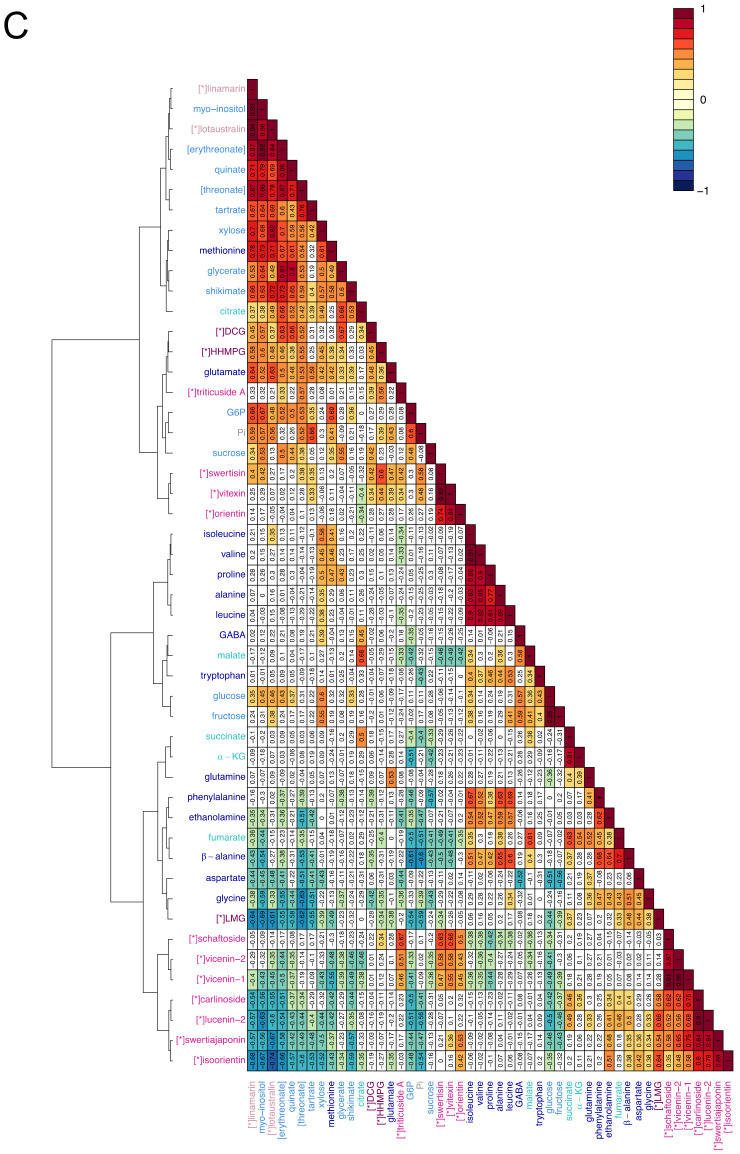
Heatmaps of the correlations between metabolites. Heatmaps were obtained *via* hierarchical cluster analysis (HCA) using the Ward clustering method. The correlation values (numbers displayed in the boxes) were calculated between couples of metabolites for the young leaf (**A**), the transition leaf (**B**), and the mature leaf (**C**) populations using Pearson correlation algorithm. Only statistically significant correlations at a correlation analysis (α = 0.05) are coloured. For every leaf sample, n = 6. The metabolites are coloured with respect to their chemical identity: amino acids and derivatives (dark blue), carbohydrates and derivatives (light blue), tricarboxylic acids (teal), cyanogenic glycosides (pink), flavonoids (fuchsia), coniferyl alcohol derivatives (plum), and inorganic compounds (grey). [*] indicates specialized metabolites.

In order to investigate the metabolic relationships, within each leaf population, the metabolites were correlated *via* hierarchical cluster analysis (HCA) using Pearson correlation algorithm and the Ward clustering method. The three heatmaps obtained (Figure 5) showed very different patterns of correlation. In the YL heatmap, two groups of metabolites stood out, one mainly composed of primary metabolites and the other of specialized metabolites (Figure 5A). The primary metabolites were highly positively correlated between each other and negatively correlated with the specialized metabolites, while strong positive correlations were observed between specialized metabolites. Globally, 77.9% of the correlations were statistically significant. Of these correlations, 14.8% had strong positive values and 6.7% had strong negative values (|0.8| < r < |1|). Among the specialized metabolites, only vitexin, isovitexin, lotaustralin, linamarin, LMG, PMG, and PDG correlated positively with primary metabolites, and negatively with the other specialized metabolites. For these compounds, the strongest positive correlation values were shown within the same chemical class (e.g., r _linamarin, lotaustralin_ = 0.96) and with shikimate (e.g., r _LMG, shikimate_ = 0.92). Shikimate itself held the highest number of positive correlations with r > 0.80. Similarly, the primary metabolites malate, citrate, glycerate, myo-inositol, and erythreonate correlated positively with the group majorly composed by specialized metabolites, and negatively with the other primary metabolites. The TL heatmap showed mainly positive and prevalently weak correlations (Figure 5B). Within the 67.6% statistically significant correlations, just 0.6% had strong negative values and 9.1% had strong positive values, of which ca. two-thirds accounted for specialized metabolites. The strongest correlation values were held by the flavonoids, except for swertisin and swertiajaponin: 63.6% of the correlations between flavonoids had r > 0.90, and 92.7% had r > 0.80. Other nearly perfect correlations were shown by glucose and fructose (r = 0.97), lotaustralin and linamarin (r = 0.94), and Pi and tartrate (r = 0.98). The latter, together with erythreonate, G6P, and malate, majorly established negative correlations with the other compounds. In the ML populations, only 43.9% of the metabolites correlated in a statistically significant way, generally with weak correlation values (Figure 5C). In fact, no correlations showed strong negative values and just 3.0% of them showed strong positive values. The latter included the correlations between flavonoids, glucose and fructose, some amino acids, linamarin, and lotaustralin.

## 3. Discussion

### 3.1. Flax Leaves Show Age-Dependent Distributions of Metabolites

Plant phenotypic plasticity is the result of the communication and inter-dependency between differently developed leaves [16], namely young leaves where growth and plastic adaptation take place and fully expanded leaves, which sustain these processes [3]. Metabolites can be particularly informative of the changes taking place within and between differently developed leaves. Firstly, being the end products of gene expression, they define the cell phenotype [1]. Secondly, relations between metabolites can shed light on the processes taking place in specific physiological conditions [17]. The purpose of this study was to obtain a snapshot of the metabolic relationships within differently developed leaves. Ultimately, this study sets the fundamentals to elucidate how these metabolic relationships are regulated when plants are facing environmental challenges. Additionally, this could provide a tool to predict the nutritional value of flax products.

In order to study the metabolic relationships characterizing flax leaves at different developmental stages, firstly, the stages had to be defined. In fact, to our knowledge, the timeline of flax development has not been previously described for a given set of parameters (e.g., light, temperature). Furthermore, because the time of development changes depending on the environmental conditions, it is indeed preferable to measure plant age taking specific physiological events as reference points [18]. For this purpose, the relative growth and expansion rates (RGR and RER) in this study were calculated for flax leaves and compared with the growth curve for dicotyledons as described by Pantin et al. [19]. Three populations of leaves were defined based on where on the growth curve the leaves were situated, namely, young (YL), transition (TL), and mature (ML). As expected, this physiological subdivision was in line with the distribution of the metabolites in the leaves. In fact, at each developmental stage leaf metabolome is the result of both the different degree of activation of the metabolic pathways and the metabolic fluxes between leaves [20]. In general, primary metabolites tended to be more concentrated in younger leaves, to support growth [21], in agreement with previous studies [22,23,24,25,26,27]. For example, in leaves of quaking aspen, similarly to flax, amino acid and G6P contents were inversely proportional with leaf age and reached their highest level in apical leaves, while carboxylic acids together with fructose and xylose were found to be prevalent in TL [23]. Specialized metabolites also showed age-dependent distribution [28,29,30]. In particular, if considered individually, they tended to be found in higher concentrations in YL, in agreement with the meta-analysis conducted by McCall and Fordyce [31]. Similar results were obtained in chicory seedlings [32]. The main phenolic compounds of flax peaked in the young leaves and their contents decreased in relation to leaf age. This could suggest a role of these molecules in plant protection against biotic or abiotic stresses. Indeed, direct defence decreases with leaf ageing [33], as expected by the optimal defence theory: the plant protects its younger leaves by endowing them with defence compounds, as they represent the most vulnerable, but also the most important organs for shoot growth and adaptation [31]. As a matter of fact, synthesis and allocation of these defence compounds must not be detrimental for the overall plant fitness. As such, for its defence, the plant uses strategies that are both inducible and constitutive. The constitutive accumulation of specialized metabolites should help plants prevent damages due to sudden herbivore attacks or environmental changes that would damage its most valuable parts. This theory was already suggested to explain the particular tissue-specific distribution pattern of phenylamides in *Nicotiana attenuata* [34]. To favour plant fitness, the contents of these metabolites must be kept to a minimum, as they are often dead-end products, as in the case for *C*-glycosyl flavonoids (discussed below). As such, the contents of specialized metabolites are usually lower than those of primary metabolites [35,36]. In this study, we only performed relative quantification. In the future, absolute quantification, provided that all standards are made available, could give information on the carbon and energy investments in the synthesis of these defence compounds. Furthermore, flux analyses or transcriptional studies may give additional clues on the site of production of the molecules (primary and specialized) and whether they are accumulated at their site of synthesis or transported between leaves of different developmental stages. In this sense, overall, the spatio-temporal distribution of the metabolites can be considered informative of the leaf physiological state.

### 3.2. Correlations between Primary Metabolites Are Indicative of the Metabolic Fluxes Between Differently Developed Leaves

Although metabolic profiles are indicative of leaf age, leaf developmental stages are conventionally established on the basis of the carbon fluxes between leaves. Accordingly, leaves are defined as source or sink leaves depending on whether they are net exporters or net importers of carbon [3]. Consequently, leaf identity can be established with certainty just through solute transport studies [37,38,39,40,41,42]. However, in this study, metabolic profiling has been proposed as a valid, less complex alternative, especially for preliminary evaluations. This is based on the use of Pearson’s correlations to investigate changes in regulation of the metabolism between different physiological states [43]. Briefly, the thorough analysis conducted by Camacho et al. [43] shows that (i) nearly perfect positive correlations are indicative of metabolites in chemical equilibrium; (ii) as a consequence, metabolites correlating negatively are not in chemical equilibrium; (iii) metabolites correlating moderately are connected by a metabolic pathway, where one of the enzymes shows high variance; and (iv) among the metabolites belonging to a moiety-conserved cycle, or other mass conservation relations, two metabolites will negatively correlate. In this analysis, absolute values of the correlation coefficient r were considered strong when r ≥ 0.8, moderate when 0.6 ≤ r ≤ 0.8, and weak when r ≤ 0.6 [44].

As a result of these considerations, each pattern of correlations can be considered as a fingerprint of the leaf population at each stage of its development [17]. The YL population was characterised by a highly contrasted profile, with primary and specialized metabolites inversely correlating between them. Primary metabolites tended to reach their highest content in YL, unlike specialized metabolites. Therefore, negative correlations between primary and specialized metabolites were to be interpreted as the preferential redirection of the available resources to the biosynthesis of primary metabolites. As such, YLs were in a state where nutrients represented the limiting resource [21]. This was expected for sink leaves, which need to support their growth with considerable amounts of nutrients, but cannot autonomously produce what they require. In particular, generally negative correlations between glucose and specialized compounds and, inversely, strong positive correlations (r > 0.80) between glucose and primary metabolites suggested that C resources were invested mainly into primary processes, most likely growth. In this regard, in leaves 61+, the flux from the amino acids toward the production of ethanolamine by the mean of serine [45], which was suggested by high correlations (r > 0.85) and high contents of ethanolamine, supported the investment of resources in the construction of cell membranes, as ethanolamine is the precursor of phospholipids [46].

TL correlation pattern showed mainly positive values, suggesting that nutrients were supplied at rates able to sustain the ensemble of the metabolic fluxes, both primary and specialized. Specifically for C, intense glycolytic flux was supported by the fact that fructose and glucose were at the chemical equilibrium (r = 0.97) [43] and that glycerate was strongly positively correlated with them (r = 0.90 with glucose, r = 0.87 with fructose). Intense metabolic activity was also suggested by high correlations between α-ketoglutarate and succinate (r = 0.90), and negative correlations between G6P and succinate (r = −0.61) and G6P and GABA (r = −0.71), as a proof that G6P derived from glucose was quickly consumed through the Krebs cycle. The greater availability of metabolites in TL relative to YL could be owing partly to the diminished growth rate, and partly to the ability to autonomously provide C compounds. In fact, flax TL leaves were assumed to be in the sink-to-source transition, a phase during which the leaf progressively acquires photosynthetic ability [39]. This was supported by the relationship between RER and degree of leaf expansion (% of final area) as reported in the timeline of leaf ontogeny for dicots [19]. Accordingly, leaf in the descendant phase of their RER curve showed leaf surface values corresponding to 30% to 60% of their final leaf surface value at full expansion. For this reason, leaves in the descendant phase of their RER curve could be defined as leaves in the sink-to-source transition [3].

Least, ML population correlation pattern showed mainly neutral or weak correlations. Fructose and glucose were still in excess (r = 0.95), but none of the previously described correlations had significant values. This supported the hypothesis that flax leaves metabolic activity decreased at later stages of development. This was also in agreement with the main role of source leaves, which is to export the hexoses produced through photosynthesis to younger metabolically active leaves.

Overall, the analysis of the metabolic correlations demonstrated that metabolic profiles can be informative on the use of C and its demand in leaves at different stages of development. This allows to create a parallel between the metabolic fingerprint of a leaf and its identity as sink, transition, or source leaf.

### 3.3. C-glycosyl Flavonoids Distribution Sheds Light on Their Biosynthetic Pathways in Flax

Previous studies have established that flax leaves are rich in *C*-glycosyl flavonoids, including vitexin, isovitexin, orientin, isoorientin, lucenin-1 and -2, and vicenin-1 and -2 [47,48,49]. Recently, our workgroup has also detected the presence of other *C*-glycosyl flavonoids in flax leaf extracts, namely, schaftoside, swertisin, carlinoside, swertiajaponin, and triticuside A [12]. In the plant kingdom, most flavonoid glycosides are *O*-glycosides, in which the sugar moiety is linked to the flavonoid skeleton via an oxygen [50]. In *C*-glycosides, on the contrary, the anomeric carbon of the sugar moiety is directly bound to the aromatic ring carbon of flavonoids through a C–C bond [50]. The strength of the *C*-glycosidic bond changes the properties of *C*-glycosyl flavonoids, as it is resistant to acid hydrolysis and glycosidase cleavage [51,52]. As a consequence, glycosylation is not transient. Moreover, the biological activity and accumulation of *C*-glycosyl flavonoids cannot be modulated (i.e., by modifying properties such as solubility and stability) [52]. This also implies that *C*-glycosides are produced just for direct use by the plant, for example, as direct defence compounds or antioxidants [53,54]. As *C*-glycosides cannot be reconverted to other biochemical pathways, they represent a cost for the plant. Therefore, their biosynthesis must be finely tuned. In most cases, expensive biosynthetic processes are limited spatially to the organs most relevant for plant fitness [10,31]. This was coherent with *C*-glycoside flavonoid distribution in flax leaves, which was inversely proportional to leaf age and directly proportional to leaf vulnerability. The content of *C*-glycosyl flavonoids decreased during development owing to inactivation or diminution of their biosynthetic enzyme activity. Decreased biosynthetic activity was also proven in citrus plants, where *C*-glycosyltransferases (CGT) transcripts were found to be more concentrated in young leaves than in mature leaves [50]. Additionally, in flax, the decrease of *C*-glycoside flavonoids may also be regarded as a phenomenon of dilution, owing to the continuous deposition of biomass during leaf structural growth. In this study, only two *C*-glycosides, namely swertisin and swertiajaponin, constituted an exception to this case. Swertisin and swertiajaponin were in fact more or less equivalently represented in YL, TL, and ML, probably because their role was not exclusively linked to young leaf protection. In this regard, it was already speculated that swertisin and swertiajaponin could act as antioxidants in oilseed flax varieties tolerant to cold [12].

The biosynthesis of *C*-glycosyl flavonoids is catalysed by *C*-glycosyltransferases (CGTs) [50]. Di-*C*-glycosides are biosynthesised from their aglycone form through two subsequent glycosylation reactions, which can be carried out by the same enzyme, as in citrus plant, or by two different enzymes, as in *Desmodium* plant [50]. In flax, the spatial distribution of mono- and di-*C*-glycosyl flavonoids seemed to support the existence of two separate enzymatic reactions. Mono-*C*-glycosides such as vitexin and isovitexin were biosynthesized very early during the leaf ontogenetic process; their concentration peaked in the youngest leaves, where it correlated positively with shikimate, which is the precursor of the phenylpropanoid pathway [55]. Di-*C*-glycosides, on the contrary, were biosynthesised at a later stage, as supported by the strong positive correlations established in TL, which was indicative of intense metabolic flux towards their production. In this regard, nearly perfect correlations were observed between schaftoside and vicenin-2, and carlinoside and lucenin-2 (r = 0.99). From the structural point of view, the compounds of these two couples differ for the presence of either glucose or arabinose at their carbon number 8. Moreover, their content is comparable throughout flax leaf development (Supplementary Materials 2). This together with high correlation values being informative of chemical equilibrium [43] suggests that schaftoside and vicenin-2 could originate from the same precursor by non-specific addition of either glucose or arabinose [50,56]. The same could happen for carlinoside and lucenin-2. In support of this hypothesis, the biosynthesis of specialized compounds is often performed by relatively non-specific enzymes in the plant kingdom [57].

## 4. Material and Methods

### 4.1. Plant Material

Flax plants were cultivated in hydroponic culture according to Quéro et al. [58]. After 31 days of growth (day 0) and during the following 10 days, the leaves were withdrawn for further analyses. At day 0, leaves 3–4, 7–8, 11–12, 15–16, 19–20, 23–24, 27–28, 31–32, 35–36, 39–40, and 43–44 were withdrawn, plus the apical bunch of leaves (leaves 45+). For the following time points (4, 7, and 10 days after the onset of the experiment), leaves 47–48, 51–52, 55–56, 57–58, and 59–60 were additionally withdrawn, plus the apical bunch of leaves (leaves 61+) (Supplementary Materials 1). Leaf samples were constituted by pooling leaves from two plants. Six replicates were collected and analysed. As the analyses performed on leaves were destructive, two populations of plants were used, one to measure the relative growth and expansion rates and the other to follow the metabolite content.

### 4.2. Leaf Dry Mass Weight and Relative Growth Rate (RGR)

After collection, leaf samples withdrawn at day 0, 4, 7, and 10 were dried in an oven at 60 °C for 48 h before being weighed on a precision scale. The rate of dry mass accumulation was followed by calculating its relative increment in time, also called the relative growth rate (RGR). The following formula was used: RGR = (ln DW_2_ − ln DW_1_)/(t_2_ − t_1_), where ln = natural logarithm, DW_1_ = dry mass weight at time one, DW_2_ = dry mass weight at time two, t_1_ = time one (in days), and t_2_ = time two (in days).

Leaf RGR was calculated between days 0–4, 4–7, and 7–10 and for leaves from 3–4 to 59–60. As leaves from 47–48 to 59–60 were too small to be handled at day 0, their dry weight values were inferred by interpolation to a curve obtained by fitting a linear trend on the dry weight values of the leaves from 27–28 to 43–44 (Supplementary Materials 3).

### 4.3. Leaf Surface and Relative Expansion Rate (RER)

Immediately after withdrawal, leaves from 3–4 to 59–60 of days 0, 4, 7, and 10 were photographed, forcing the flash to even the external light conditions. The images were treated using the software Image J to measure the leaf surface. The rate of leaf expansion was further monitored by calculating the relative expansion rate (RER), according to the following formula: RER = (ln LS_2_ – ln LS_1_)/(t_2_ – t_1_), where ln = natural logarithm, LS_1_ = leaf surface at time one, LS_2_ = leaf surface at time two t_1_ = time one (in days), and t_2_ = time two (in days).

As leaves from 43–44 to 59–60 were too small to be handled at day 0, their leaf surface values were inferred by interpolation to a curve obtained by fitting a linear trend on the leaf surface values of the leaves from 23–24 to 39–40 (Supplementary Materials 3).

### 4.4. Metabolic Profiling

Leaves withdrawn at day 4 underwent metabolic profiling. Polar metabolites were extracted with a mixture of methanol, chloroform, and water (2:1:2; V:V:V), as described by Quéro et al. [59]. Profiling of primary metabolites by gas chromatography coupled to mass spectrometry (GC-MS) analysis was conducted according to Quéro et al. [59]. The results are expressed in area ratio/g of DW. The area ratio corresponds to the area of each metabolite divided by the area of the internal standard (ribitol). A representative chromatogram and a detailed protocol for GC-MS analysis can be found in Supplementary Materials 4. The profiling of specialized metabolites by ultra-performance liquid chromatography coupled to mass spectrometry (UPLC-MS) (Waters, Manchester, UK) analysis was performed as described by Tchoumtchoua et al. [12]. The results are expressed in area/g of DW. A representative chromatogram and a detailed protocol for LC-MS can be found in Supplementary Materials 5.

### 4.5. Data Treatment

#### 4.5.1. Statistical Analysis

For each metabolite, area ratio (AR) values of the different leaf samples were compared using a Kruskal–Wallis analysis (*p* < 0.05, *p*-adjusted Bonferroni) run in R software (v. 3.6.1, company Foundation for Statistical Computing, Vienna, Austria) using the function “kruskal” of the package “agricolae”.

#### 4.5.2. Multivariate Analysis

Multivariate data treatment was performed by the mean of principal component analysis (PCA) using the software SIMCA (V 14.1, Umetrics). The data were unit variance (UV) scaled.

#### 4.5.3. Kohonen Self-Organising Map (SOM)

The unsupervised self-organizing map (SOM) algorithm was run on the data matrix constituted by the values of content (area ratio (AR) or area (A) per g of dry matter) of the leaf sample metabolites. For this purpose, the packages “som” and “kohonen” were used in R software (v. 3.6.1, company Foundation for Statistical Computing, Vienna, Austria). For each metabolite, data were first normalised by expressing the content values of each leaf sample as a percentage of the higher content shown by the metabolite. The function “som” was used to project the data in a matrix with 4 × 5 nodes, of hexagonal topology, Gaussian neighbourhood, and rlen = 10,000 (the number of times the complete data set was presented to the network). For their representation (Figure 4), the SOM data were unit variance (UV) scaled. This allowed to calculate the standard deviation of the content of each metabolite of a class from the content of the class itself for every leaf sample.

#### 4.5.4. Hierarchical Cluster Analysis (HCA)

Correlations between metabolites were calculated and visualised through hierarchical cluster analysis (HCA) in R software (v. 3.6.1, company Foundation for Statistical Computing, Vienna, Austria) using the functions of the package “mixOmics”. Pearson linear correlation values were calculated using the function “cor” (method = c(“pearson”)). Statistically significant correlations were defined with the aid of the “cor.test” function (α = 0.05, method = c(“pearson”)). The statistically significant correlations were then visualised in a colour-coded cluster image map (CIM). The CIM was produced by running the “cim” algorithm on the correlation matrix previously created, using the Ward clustering method (clust.method = c(“ward”,“ward”)). HCA was performed separately for populations of young (leaves 51–52 to 61+), transition (leaves from 27–28 to 47–48), or mature (leaves from 3–4 to 23–24) leaves.

## 5. Conclusions

In this study, metabolic profiling was used to investigate primary and specialized metabolite distribution in flax leaves. Metabolite levels varied along the shoot in an age-dependent fashion, with the majority of the metabolites being concentrated in the youngest, apical leaves. Leaf metabolic composition was also revealed to be able to discriminate leaves in different phases of their growth. This tool may be useful to assess leaf age when the timeline of the leaf ontogeny in specific environmental conditions is not known. Visualisation of the metabolic fluxes using correlation heatmaps suggested that the metabolic identity of differentially-developed leaves was influenced by the availability of carbon resources. This finding reinforced the use of metabolic profiling as a tool to discriminate sink, transition, and source leaves. The global analysis of our data also allowed us to link plant specialized metabolite distribution, namely *C*-glycosyl flavonoids, with primary metabolite availability, showing that their degree of glycosylation is age-dependent. Overall, this study will serve as a basis for investigating how external cues can affect the metabolic identity of differently-developed organs and their metabolic relationships, with the aim of targeting processes for the amelioration of flax crop productivity. In the future, this study could also be extended to the flowering stages. Prediction of resource allocation (of both polar and apolar compounds) towards the developing seeds in relation to external or internal cues could help to predict and improve the nutritional values of this high value crop product.

## Supplementary Materials

The following are available online at https://www.mdpi.com/2218-1989/10/6/218/s1, Figure S1: Withdrawal of flax leaves; Figure S2: Metabolite content of flax leaves; Figure S3: Inference of dry weight and leaf surface values of leaves that could not be withdrawn at day 0; Figure S4: Representative chromatogram and a detailed protocol for GC-MS analysis; Figure S5: Representative chromatogram and a detailed protocol for LC-MS analysis.

## References

[1] Sumner L.W., Mendes P., Dixon R.A. (2003). Plant metabolomics: Large-scale phytochemistry in the functional genomics era. Phytochemistry.

[2] Kehr J. (2001). High resolution spatial analysis of plant systems. Curr. Opin. Plant Boil..

[3] Turgeon R. (1989). The sink-source transition in leaves. Annu. Rev. Plant Physiol. Plant Mol Biol..

[4] Fatichi S., Leuzinger S., Körner C. (2014). Moving beyond photosynthesis: From carbon source to sink-driven vegetation modeling. New Phytol..

[5] Blum A. (2017). Osmotic adjustment is a prime drought stress adaptive engine in support of plant production. Plant Cell Environ..

[6] Sultan S.E. (2000). Phenotypic plasticity for plant development, function and life history. Trends Plant Sci..

[7] Yu S.-M., Lo S.-F., Ho T.-H.D. (2015). Source-sink communication: Regulated by hormone, nutrient, and stress cross-signaling. Trends Plant Sci..

[8] Wink M. (1988). Plant breeding: Importance of plant secondary metabolites for protection against pathogens and herbivores. Theoret. Appl. Genetics.

[9] Kim J.K., Park S.-Y., Lim S.-H., Yeo Y., Cho H.S., Ha S.-H. (2013). Comparative metabolic profiling of pigmented rice (*Oryza sativa* L.) cultivars reveals primary metabolites are correlated with secondary metabolites. J. Cereal Sci..

[10] Koricheva J. (2002). Meta-analysis of sources of variation in fitness costs of plant antiherbivore defenses. Ecology.

[11] Goyal A., Sharma V., Upadhyay N., Gill S., Sihag M. (2014). Flax and flaxseed oil: An ancient medicine & modern functional food. J. Food Sci. Technol..

[12] Tchoumtchoua J., Mathiron D., Pontarin N., Gagneul D., van Bohemen A.-I., Otogo N’nang E., Mesnard F., Petit E., Fontaine J.-X., Molinié R. (2019). Phenolic profiling of flax highlights contrasting patterns in winter and spring varieties. Molecules.

[13] Schwachtje J., Baldwin I.T. (2008). Why does herbivore attack reconfigure primary metabolism?. Plant Physiol..

[14] Aharoni A., Galili G. (2011). Metabolic engineering of the plant primary-secondary metabolism interface. Curr. Opin. Biotechnol..

[15] Kim H.K., Choi Y.H., Verpoorte R., Saito K., Dixon R.A., Willmitzer L. (2006). Metabolomic analysis of *Catharanthus roseus* using NMR and principal component analysis. Plant Metabolomics.

[16] Weiner J. (2004). Allocation, plasticity and allometry in plants. Perspect. Plant Ecol. Evol. Syst..

[17] Steuer R. (2006). Review: On the analysis and interpretation of correlations in metabolomic data. Brief Bioinform..

[18] Erickson R.O., Michelini F.J. (1957). The plastochron index. Am. J. Bot..

[19] Pantin F., Simonneau T., Muller B. (2012). Coming of leaf age: Control of growth by hydraulics and metabolics during leaf ontogeny. New Phytol..

[20] Freitas J.R.L., Vendramini P.H., Melo J.O.F., Eberlin M.N., Augusti R. (2018). An appraisal on the source-to-sink relationship in plants: An application of desorption electrospray ionization mass spectrometry imaging. J. Braz. Chem. Soc..

[21] Bénard C., Bernillon S., Biais B., Osorio S., Maucourt M., Ballias P., Deborde C., Colombié S., Cabasson C., Jacob D. (2015). Metabolomic profiling in tomato reveals diel compositional changes in fruit affected by source-sink relationships. J. Exp. Bot..

[22] Masclaux C., Valadier M.H., Brugière N., Morot-Gaudry J.F., Hirel B. (2000). Characterization of the sink/source transition in tobacco (*Nicotiana tabacum* L.) shoots in relation to nitrogen management and leaf senescence. Planta.

[23] Jeong M.L., Jiang H., Chen H.-S., Tsai C.-J., Harding S.A. (2004). Metabolic profiling of the sink-to-source transition in developing leaves of quaking aspen. Plant Physiol..

[24] Desbrosses G.G., Kopka J., Udvardi M.K. (2005). *Lotus japonicus* metabolic profiling. Development of gas chromatography-mass spectrometry resources for the study of plant-microbe interactions. Plant Physiol..

[25] Kusano M., Jonsson P., Fukushima A., Gullberg J., Sjöström M., Trygg J., Moritz T. (2011). Metabolite signature during short-day induced growth cessation in *Populus*. Front. Plant Sci..

[26] Fester T., Fetzer I., Härtig C. (2013). A core set of metabolite sink/source ratios indicative for plant organ productivity in *Lotus japonicus*. Planta.

[27] Watanabe M., Balazadeh S., Tohge T., Erban A., Giavalisco P., Kopka J., Mueller-Roeber B., Fernie A.R., Hoefgen R. (2013). Comprehensive dissection of spatiotemporal metabolic shifts in primary, secondary, and lipid metabolism during developmental senescence in Arabidopsis. Plant Physiol..

[28] Kleiner K.W., Raffa K.F., Dickson R.E. (1999). Partitioning of ^14^C-labeled photosynthate to allelochemicals and primary metabolites in source and sink leaves of aspen: Evidence for secondary metabolite turnover. Oecologia.

[29] Matsuki S., Sano Y., Koike T. (2004). Chemical and physical defence in early and late leaves in three heterophyllous birch species native to northern Japan. Ann. bot..

[30] Matsuda F., Hirai M.Y., Sasaki E., Akiyama K., Yonekura-Sakakibara K., Provart N.J., Sakurai T., Shimada Y., Saito K. (2010). AtMetExpress development: A phytochemical atlas of Arabidopsis development. Plant Physiol..

[31] McCall A.C., Fordyce J.A. (2010). Can optimal defence theory be used to predict the distribution of plant chemical defences?. J. Ecol..

[32] Legrand G., Delporte M., Khelifi C., Harant A., Vuylsteker C., Mörchen M., Hance P., Hilbert J.-L., Gagneul D. (2016). Identification and characterization of five BAHD acyltransferases involved in hydroxycinnamoyl ester metabolism in chicory. Front. Plant Sci..

[33] Yamawo A., Suzuki N., Tagawa J., Hada Y. (2012). Leaf ageing promotes the shift in defence tactics in *Mallotus japonicus* from direct to indirect defence. J. Ecol..

[34] Kaur H., Heinzel N., Schöttner M., Baldwin I.T., Gális I. (2010). R2R3-NaMYB8 Regulates the accumulation of phenylpropanoid-polyamine conjugates, which are essential for local and systemic defense against insect herbivores in *Nicotiana attenuata*. Plant Physiology.

[35] Isah T. (2019). Stress and defense responses in plant secondary metabolites production. Biol. Res..

[36] Ncube B., Van Staden J. (2015). Tilting plant metabolism for improved metabolite biosynthesis and enhanced human benefit. Molecules.

[37] Jones H., Eagles J.E. (1962). Translocation of ^14^carbon within and between leaves. Ann. bot..

[38] Fellows R.J., Geiger D.R. (1974). Structural and physiological changes in sugar beet leaves during sink to source conversion. Plant Physiol..

[39] Roberts A.G., Cruz S.S., Roberts I.M., Prior D., Turgeon R., Oparka K.J. (1997). Phloem unloading in sink leaves of *Nicotiana benthamiana*: Comparison of a fluorescent solute with a fluorescent virus. Plant Cell.

[40] Imlau A., Truernit E., Sauer N. (1999). Cell-to-cell and long-distance trafficking of the green fluorescent protein in the phloem and symplastic unloading of the protein into sink tissues. Plant Cell.

[41] Meng Q., Siebke K., Lippert P., Baur B., Mukherjee U., Weis E. (2001). Sink–source transition in tobacco leaves visualized using chlorophyll fluorescence imaging. New Phytol..

[42] Merry A.M., Evans K.J., Corkrey R., Wilson S.J. (2013). Coincidence of maximum severity of powdery mildew on grape leaves and the carbohydrate sink-to-source transition. Plant Pathol..

[43] Camacho D., de la Fuente A., Mendes P. (2005). The origin of correlations in metabolomics data. Metabolomics.

[44] Weckwerth W., Loureiro M.E., Wenzel K., Fiehn O. (2004). Differential metabolic networks unravel the effects of silent plant phenotypes. Proc. Natl. Acad. Sci. USA.

[45] Guschina I.A., Everard J.D., Kinney A.J., Quant P.A., Harwood J.L. (2014). Studies on the regulation of lipid biosynthesis in plants: Application of control analysis to soybean. Biochim. Biophys. Acta.

[46] Voet D., Voet J.G., Pratt C.W. (2016). Fundamentals of biochemistry: Life at the molecular level.

[47] Ibrahim R.K. (1969). Chromatographic and spectrophotometric evidence for the occurrence of mixed *O*- and *C*-glycoflavones in flax (*Linum usitatissimum*) cotyledons. Biochim. Biophys. Acta.

[48] Dubois J., Mabry T.J. (1971). The *C*-glycosylflavonoids of flax, *Linum usitatissimum*. Phytochemistry.

[49] Czemplik M., Mierziak J., Szopa J., Kulma A. (2016). Flavonoid *C*-glucosides derived from flax straw extracts reduce human breast cancer cell growth *in vitro* and induce apoptosis. Front. Pharmacol..

[50] Ito T., Fujimoto S., Suito F., Shimosaka M., Taguchi G. (2017). *C*-Glycosyltransferases catalyzing the formation of di-*C*-glucosyl flavonoids in citrus plants. Plant J..

[51] Talhi O., Silva A.M.S. (2012). Advances in *C*-glycosylflavonoid research. Curr. Org. Chem..

[52] Xiao J., Capanoglu E., Jassbi A.R., Miron A. (2016). Advance on the flavonoid *C*-glycosides and health benefits. Crit Rev Food Sci Nutr.

[53] Pietta P.G. (2000). Flavonoids as antioxidants. J. Nat. Prod..

[54] McNally D.J., Wurms K.V., Labbé C., Quideau S., Bélanger R.R. (2003). Complex *C*-glycosyl flavonoid phytoalexins from *Cucumis sativus*. J. Nat. Prod..

[55] Vogt T. (2010). Phenylpropanoid biosynthesis. Mol. Plant.

[56] Hamilton M.L., Kuate S.P., Brazier-Hicks M., Caulfield J.C., Rose R., Edwards R., Torto B., Pickett J.A., Hooper A.M. (2012). Elucidation of the biosynthesis of the di-*C*-glycosylflavone isoschaftoside, an allelopathic component from *Desmodium* spp. that inhibits *Striga* spp. development. Phytochemistry.

[57] Schwab W. (2003). Metabolome diversity: Too few genes, too many metabolites?. Phytochemistry.

[58] Quéro A., Molinié R., Elboutachfaiti R., Petit E., Pau-Roblot C., Guillot X., Mesnard F., Courtois J. (2014). Osmotic stress alters the balance between organic and inorganic solutes in flax (*Linum usitatissimum*). J. Plant Physiol..

[59] Quéro A., Fliniaux O., Elboutachfaiti R., Petit E., Guillot X., Hawkins S., Courtois J., Mesnard F. (2015). β-Aminobutyric acid increases drought tolerance and reorganizes solute content and water homeostasis in flax (*Linum usitatissimum*). Metabolomics.

