# Supplementary material for: Age-Dependent Metabolic Profiles Unravel the Metabolic Relationships within and between Flax Leaves (Linum usitatissimum)
Source: Metabolites. 2020 May 26;10(6):218. doi: 10.3390/metabo10060218 (PMC7345097; doi:10.3390/metabo10060218)
Supplement: Supplementary file 1 [file metabolites-10-00218-s001.pdf]

Supplementary materials 1. Withdrawal of leaves from flax plants at the stage 15 cm cultivated in hydroponic culture. Pairs of leaves were withdrawn alternatively from leaves 3-4 until leaves 55-56. Then, leaves 55-56, 57-58, 59-60 and 61+ were withdrawn. In the figure, leaves 61+ correspond to the apical leaves. The colours indicate leaves at different developmental stages, namely young (green), transition (yellow) and mature (red) leaves. The position of the first leaf sample of each developmental stage is shown. In detail, leaves 3-4 are the first leaves of the mature leaf sample population, leaves 27-28 those of the transition one and leaves 51-52 those of the young one. At the right of the names of the leaf samples, the images clarify that the samples were constituted by 4 leaves (two leaf pairs, each from one of the two plants of the same biological replicate), with the exception of the apical leaves which were withdrawn together because of their small size. For this reason, the number of the apical leaves was variable between biological replicates.

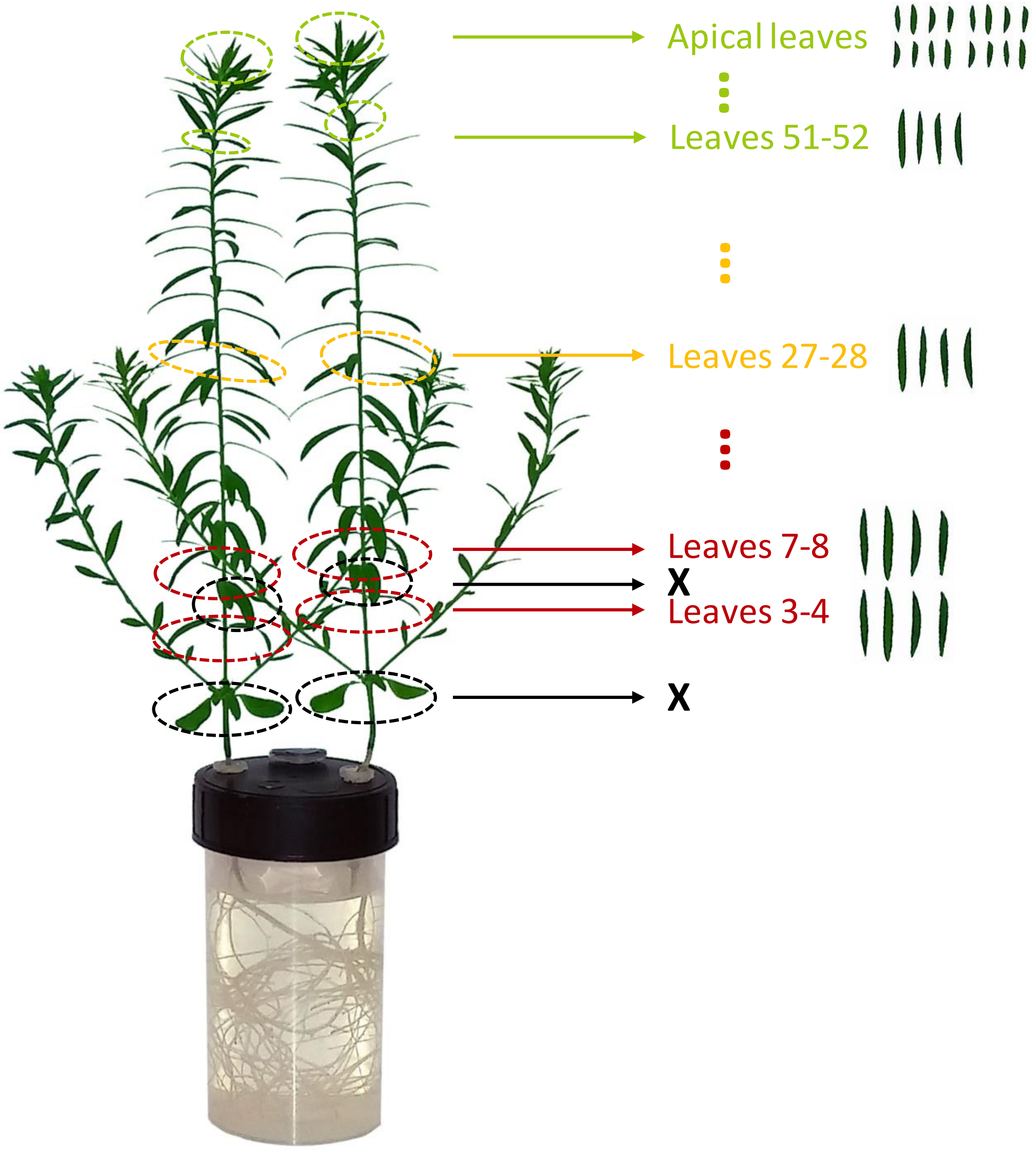

Supplementary materials 2. Metabolic content of flax leaves. Each bar of the histogram represents either the peak area (A) or the area ratio between the peak areas of the compound and the internal standard (AR) per g f dry weight (DW). For each metabolite, leaves showing statistically significant different metabolic contents are indicated by different letters (Kruskal-Wallis analysis, P<0.05, P adjusted Bonferroni, n=6). The compounds indicated in brackets were identified by matching their mass spectra and retention index (RI) to the data from the Golm Metabolome Database. All the other compounds were identified in comparison to pure standards.

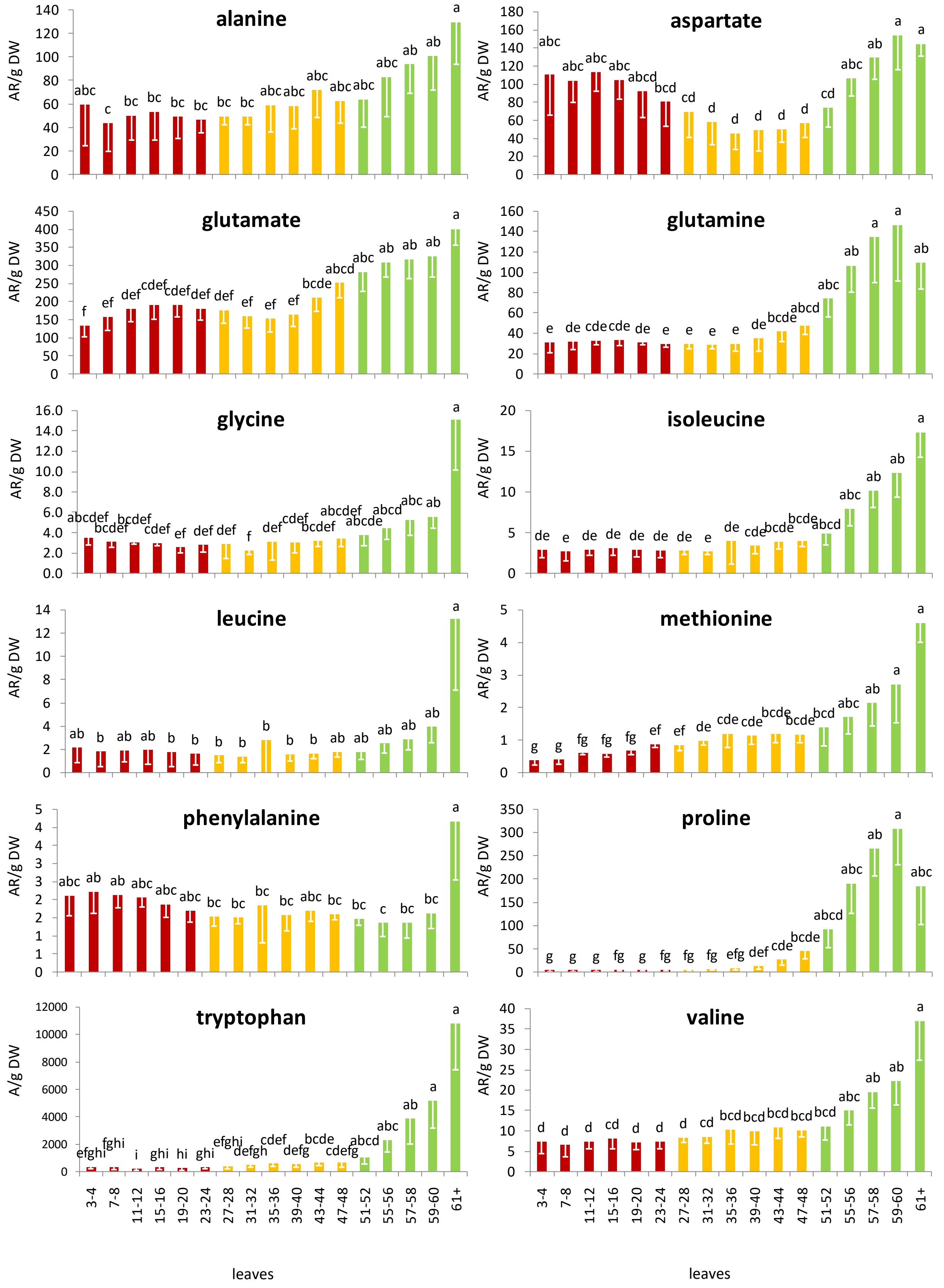

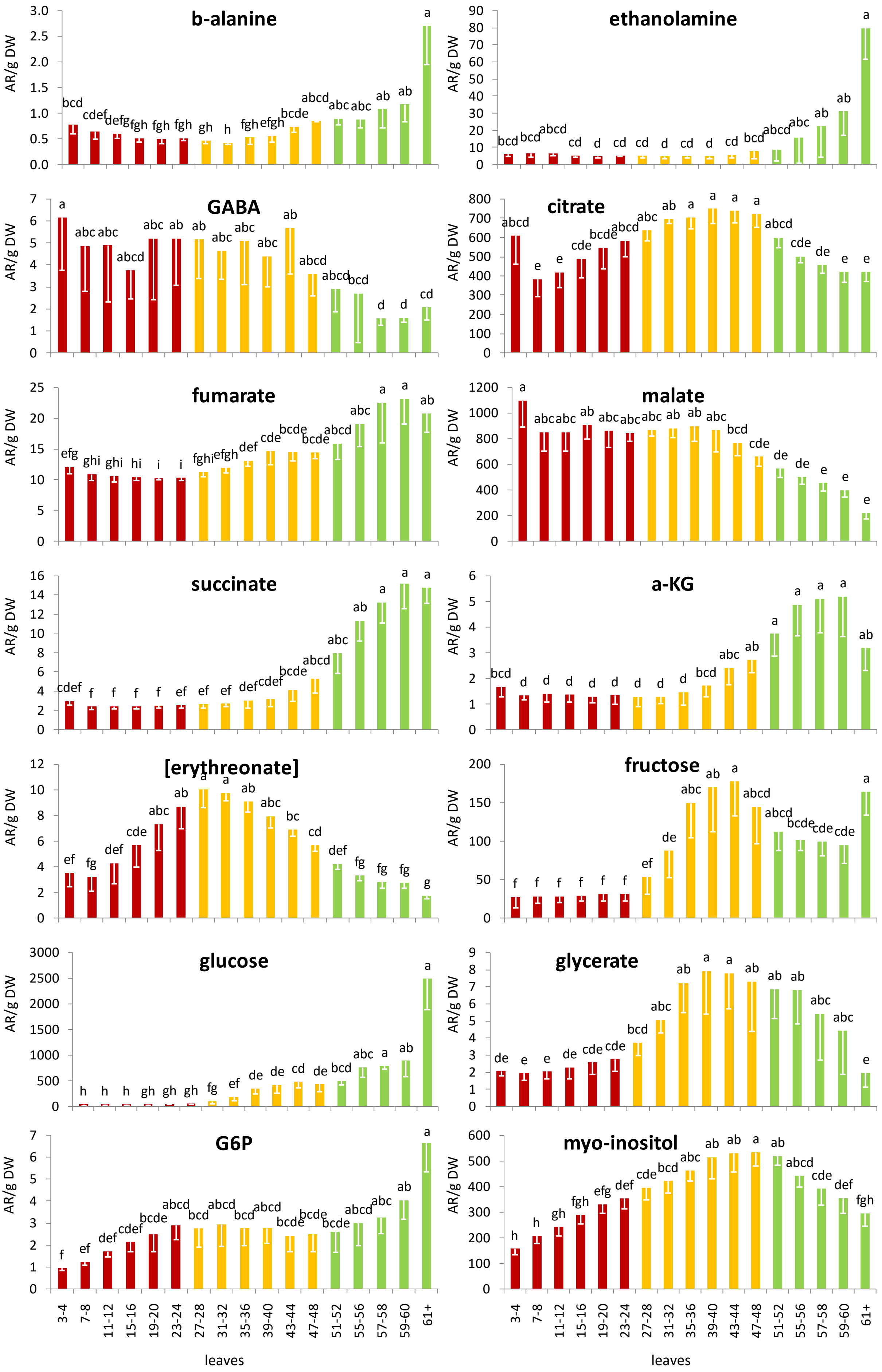

b-alanine
ethanolamine
GABA
citrate
fumarate
malate
succinate
a-KG
[erythreonate]
fructose
glucose
glycerate
G6P
myo-inositol
AR/g DW
3-4
7-8
11-12
15-16
19-20
23-24
27-28
31-32
35-36
39-40
43-44
47-48
51-52
55-56
57-58
59-60
61+
leaves

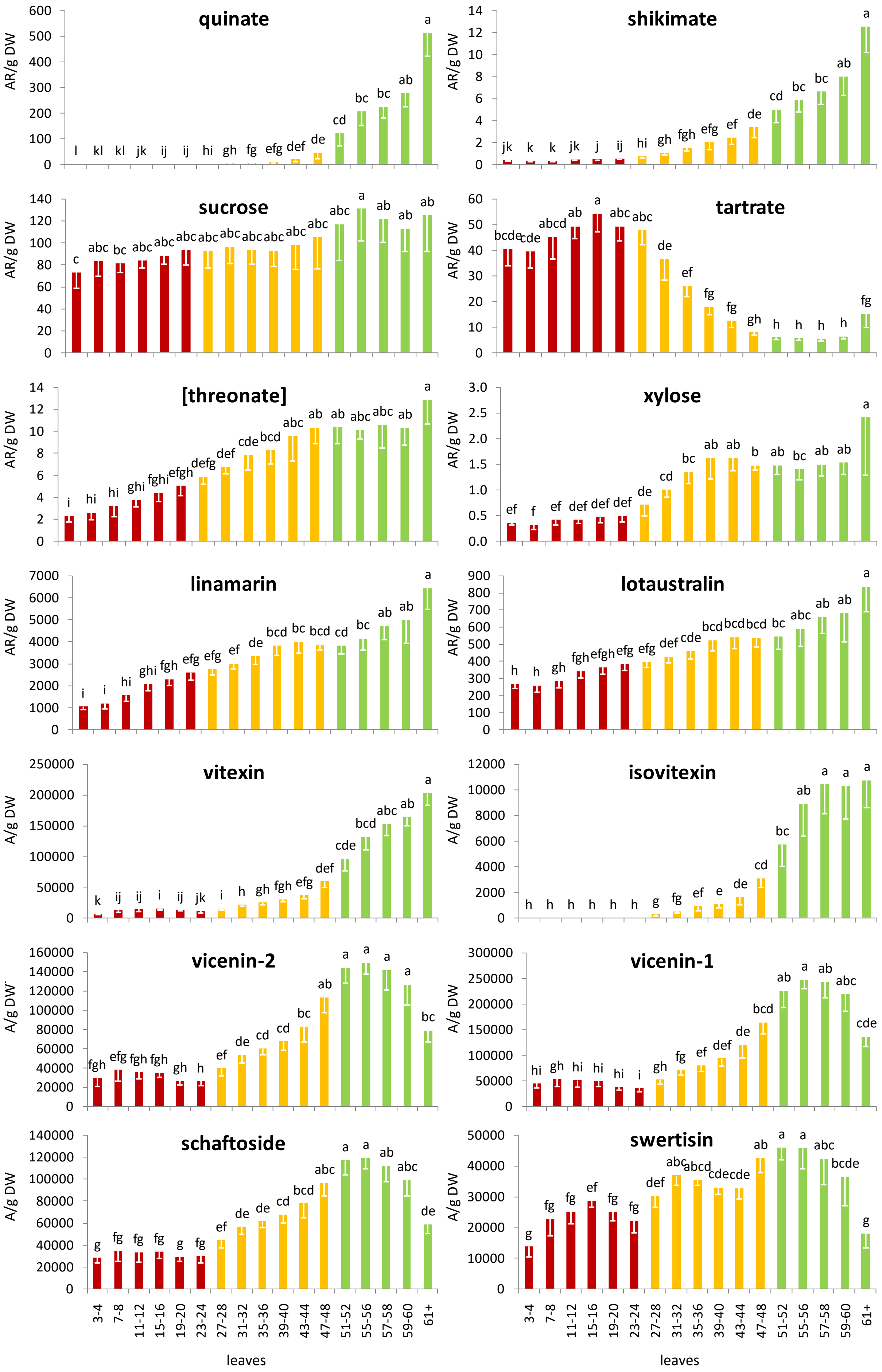
quinate
shikimate
sucrose
tartrate
[threonate]
xylose
linamarin
lotaustralin
vitexin
isovitexin
vicenin-2
vicenin-1
schaftoside
swertisin
AR/g DW
A/g DW
3-4
7-8
11-12
15-16
19-20
23-24
27-28
31-32
35-36
39-40
43-44
47-48
51-52
55-56
57-58
59-60
61+
leaves

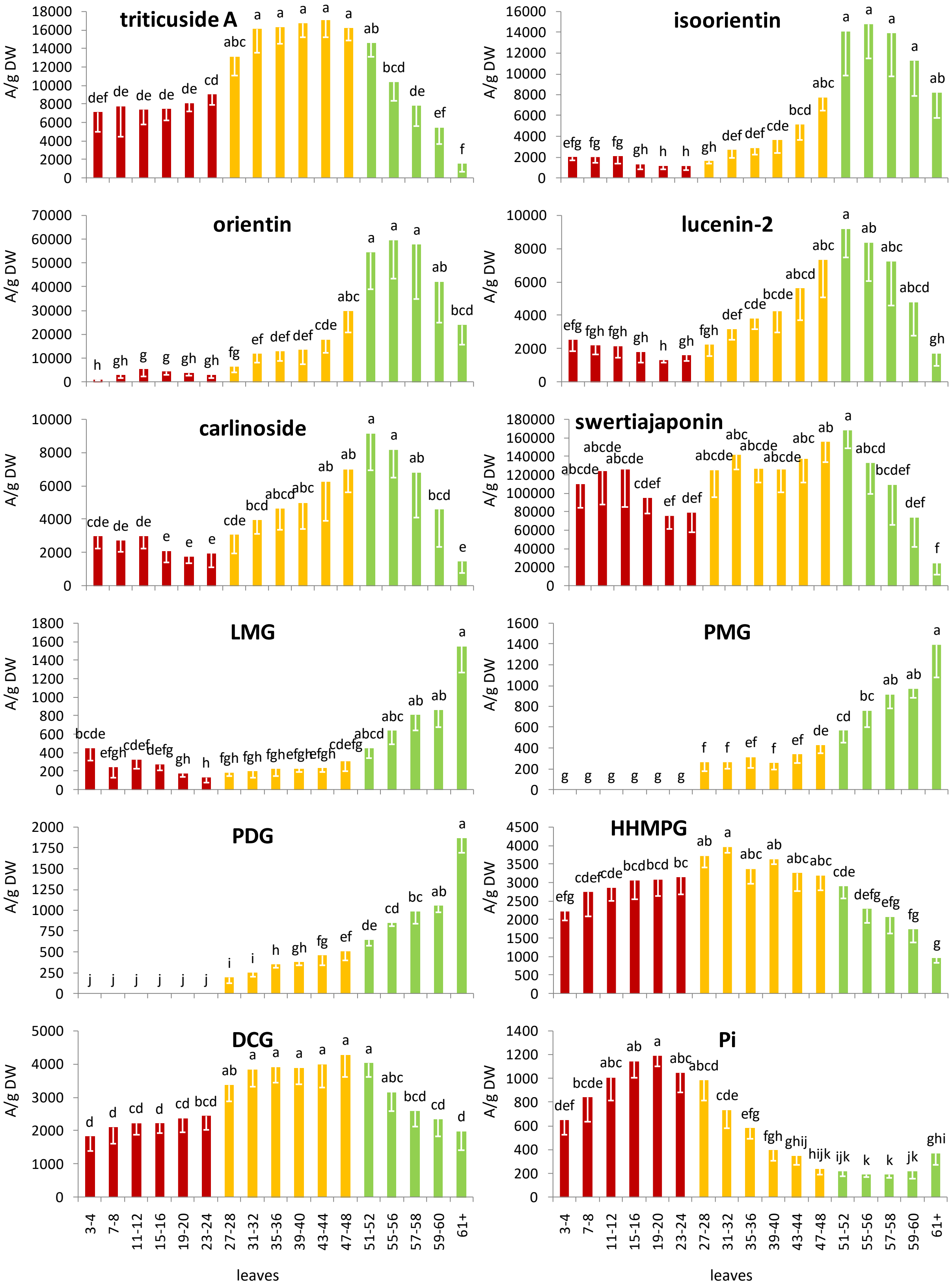
triticuside A
isoorientin
orientin
lucenin-2
carlinoside
swertiajaponin
LMG
PMG
PDG
HHMPG
DCG
Pi
A/g DW
3-4
7-8
11-12
15-16
19-20
23-24
27-28
31-32
35-36
39-40
43-44
47-48
51-52
55-56
57-58
59-60
61+
leaves

Supplementary materials 3. Inference of dry weight and leaf surface values of leaves that could not be withdrawn at day 0. Dry weight values of leaves from 47-48 to 59-60 (empty dots) (left) and leaf surface values of leaves from 43-44 to 59-60 (empty dots) (right) were calculated by interpolation to a curve obtained by fitting a linear trend on the values of the leaves from 27-28 to 43-44 for dry weight and from 23-24 to 39-40 for leaf surface (full dots, n=6). R^2 : coefficient of determination.

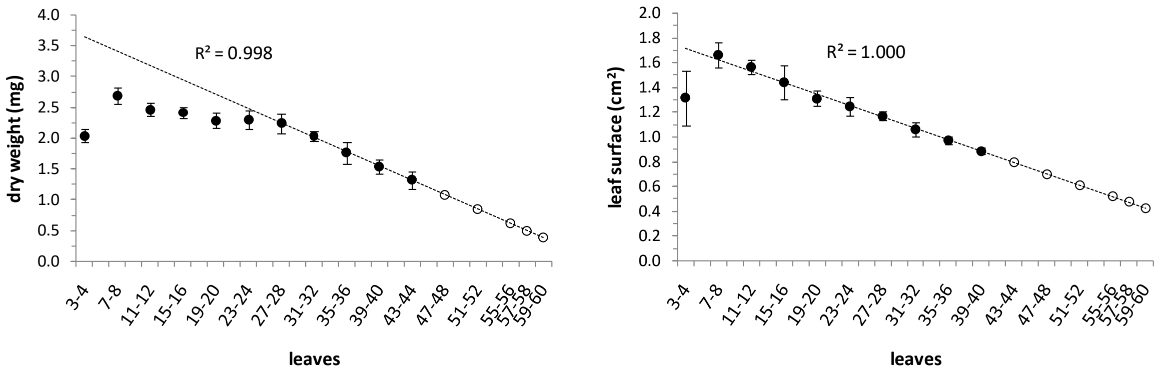

Supplementary materials 4. Representative chromatogram and detailed protocol for GC-MS analysis. Chromatograms of mature leaves (red), transition leaves (yellow) and young leaves (green) obtained by GC-MS analysis. 2: aspartate 3TMS, 3: glutamate 3TMS, 4 : glutamine 3TMS, 10: proline 2TMS, 16 : citrate 4TMS, 18: malate 3TMS, 19: succinate 2TMS, 22: fructose 2 Meox 5TMS, 23: glucose 1Meox 5TMS, 26: myo-inositol 6TMS, 27: quinate 5TMS, 29: sucrose 8TMS, 33: linamarin 4TMS, 34 : lotaustralin 4TMS, 52 :Pi 3TMS. IS: Internal Standard (ribitol 5TMS), Meox: Methoxyamine, TMS: Trimethylsilyl. The signals of alanine, glycine, isoleucine, leucine, methionine, phenylalanine, tryptophan, valine, β-alanine, ethanolamine, GABA, fumarate, α-KG, erythreonate, glycerate, G6P, shikimate, tartrate, threonate, xylose were too low to be indicated on the chromatogram.

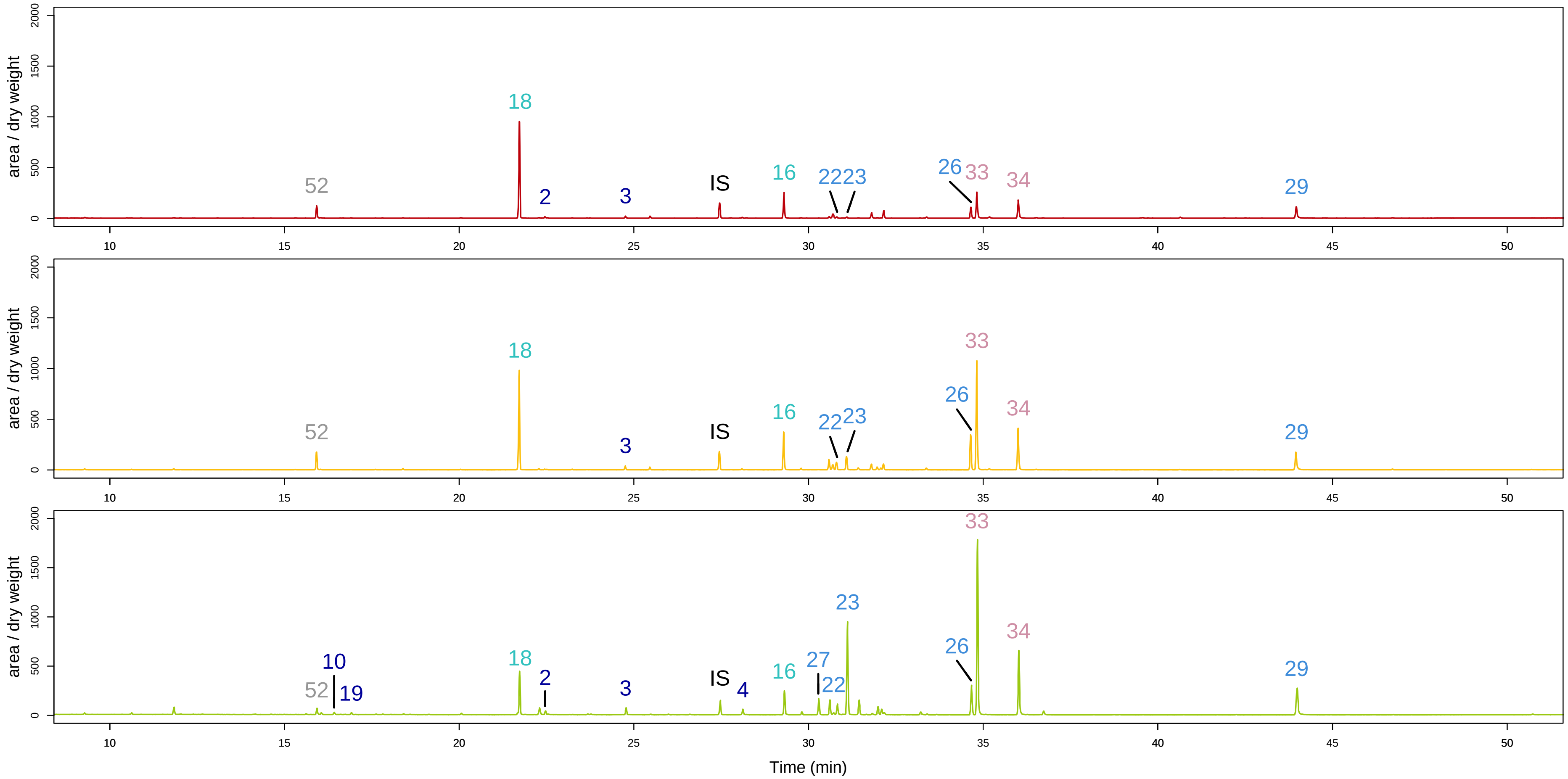

Protocol for GC-MS analysis: Before GC-MS analysis a step of derivatization (trimethylsilylation and methoxylation) was performed as described hereafter. Fifty µL of the raw extract were dried in a vacuum concentrator and afterward supplemented with 40 µL of pyridine methoxyamine solution (20 mg/mL) (Sigma-Aldrich). The solution was stirred in a stirring dry incubator at 950 rpm for 2 h at 37°C. A short centrifugation followed (6 sec) aimed at collecting the solution at the bottom of the microtube. Subsequently, 70 µL of *N*-methyl-*N*-(trimethylsilyl)trifluoroacetamide (MSTFA) (Sigma-Aldrich) were added before stirring again at 950 rpm for 30 min at 37°C. At the end of the reaction, the samples were again shortly centrifuged and then transferred in glass vials to be either directly analysed by GC-MS or stored at -20°C before analysis.

Profiling of primary metabolites was performed on a TRACE GC ULTRA gas chromatograph coupled to a DSQII quadrupole mass spectrometer (Thermo Fisher Scientific). For the chromatographic separation, a TraceGOLD TG-5MS GC capillary column (Thermo Fisher Scientific) was used (5 % phenyl matrix, 30 m x 0.25 mm x 0.25 µm). Helium was chosen as a carrier gas at a flux of 1 mL/min.

A volume of 1 µL of sample was injected with a split ratio of 25:1. The injection temperature was 230°C to allow a quick and complete volatilisation of the compounds of the sample matrix. The temperature program was set for the first 5 min at 70°C, followed by a ramp of 5°C/min until reaching 310°C, which temperature was maintained for 3 min at the end.

Concerning the mass spectrometry analysis, the temperature of the interface between the GC and the MS was set to 280°C, and the one of the ion volume at 220°C. Mass spectra were recorded at 2 scans/sec in a range of 50 to 600 *m*/*z*. Due to the large number of samples, the analytical conditions were verified by making use of a Quality Control (QC) sample, whose analysis was interposed to every 5 analysed samples. The QC was constituted by pooling a representative population of the analysed samples.

The chromatographic peaks and the mass spectra were visualised through the software Xcalibur (Thermo Scientific). The compounds considered for metabolic profiling had been previously identified on the basis of their retention time and their mass spectrum. Briefly, the identification made use of the software Automated Mass Spectral Deconvolution and Identification System (AMDIS) and the online library Golm Metabolome Database (http://gmd.mpimp-golm.mpg.de/search.aspx). In this phase, the metabolites were assigned an identity by comparison of mass spectra. The chemical nature of the majority of the metabolites was afterward confirmed by analysing the corresponding analytical standards. Finally, the metabolites were quantified from a quantification ion which was chosen with respect to its relative intensity and for its specificity to the compound. This last characteristic was used to maximally limit any wrong estimation due to co-elution.

Supplementary materials 5. Representative chromatogram and detailed protocol for LC-MS analysis. Chromatograms of mature leaves (red), transition leaves (yellow) and young leaves (green) obtained by LC-MS analysis. 35: vitexin, 37: vicenin-2, 38: vicenin-1, 39: schaftoside, 40: swertisin, 42: isoorientin, 43: orientin, 46: swertiajaponin. The signals of isovitexin, triticusideA, lucenin-2, carlinoside, LMG, PMG, PDG, HHMPG, DCG were too low to be indicated on the chromatogram.

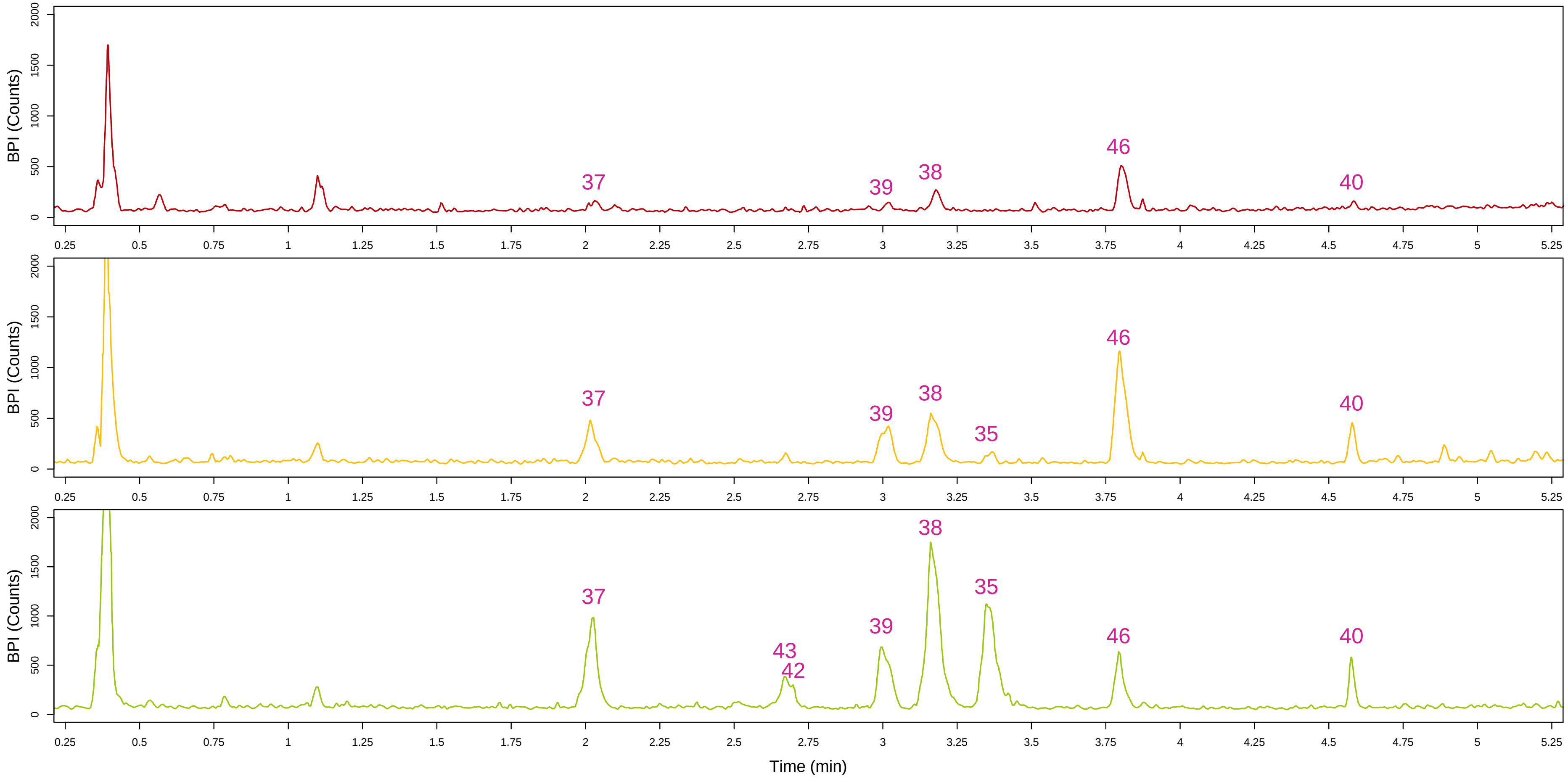

Protocol for LC-MS analysis: UPLC-MS analysis was carried on an ACQUITY UPLC I-Class system (Waters) coupled to a Vion IMS QTof (Ion Mobility Quadrupole Time-of-flight) hybrid mass spectrometer, equipped with an electrospray ionization (ESI) source (Waters). One µL of each sample was injected. The elution was performed on a Kinetex Biphenyl (100 mm x 2.1 mm x 1.7 µm) column (Phenomenex), maintained at 55°C. The mobile phase flux was set to 0.55 mL/min and programmed to a gradient going from water with formic acid 0.1 % (A) to methanol with formic acid 0.1 % (B) as follows (A:B): 80:20 (t=0min), 80:20 (t=0.5min), 40:60 (t=5min), 10:90 (t=6 min), 10:90 (t=7 min), 80:20 (t=7.5min), 80:20 (t=10 min). The ESI source was set to a capillary voltage of 2.5 kV for the negative mode, a source temperature of 120°C and a desolvation temperature of 450°C. The TOF was operated in the sensitivity mode, providing an average resolving power of approximately 50,000 (FWHM) in the scanning 50-1200 *m/z* range and a scan time of 0.2 s. Data acquisition and data treatment was performed with UNIFI software (V1.8, Waters). QC samples were analysed every 10 analysed samples to verify the reproducibility of the analytical conditions in time. All the analyzed compounds have been identified on the basis of their retention time and mass spectrum, by comparison with commercial standard or with compounds purified from flax leaves by preparative HPLC and then characterized by LC-MS and NMR in the laboratory.
